# O5-3 Behavioural risk patterns among overweight and obese adolescents participating in the PRALIMAP-INES trial

**DOI:** 10.1093/eurpub/ckac094.035

**Published:** 2022-08-29

**Authors:** Chantal Julia, Abdou Omorou, Francis Guillemin, Serge Briançon

**Affiliations:** 1 Université Paris 13, CRESS, INSERM, INRA, Cnam, Paris, France; 2 CHRU de Nancy, Vandoeuvre-Lès-Nancy, France; 3 Nancy Public Health School, University of Lorraine, Vandoeuvre-Lès-Nancy, France; 4 Apemac, University of Lorraine, Vandoeuvre-Lès-Nancy, France

**Keywords:** Adolescents, Overweight and obesity, Physical activity, Sedentary behavior

## Abstract

**Background:**

It has been suggested that risk behaviours including diet, physical activity (PA), sedentary behaviour (SB), smoking and alcohol consumption cluster among adolescents. Our objective was to investigate clustering of risk behaviours in overweight and obese adolescents from the French PRALIMAP-InèS trial and to identify their socioeconomic correlates.

**Methods:**

Information on diet (fruit, vegetables, sugary products and beverages), PA, SB (week and week-end days), smoking and alcohol consumption (current frequency and intoxication episodes) and socio-demographical data were collected using self-reported questionnaires at the trial inclusion. Behavioural risk factors were entered as categorical variables in a two-step clustering procedure: multiple correspondence analyses followed by a two-way clustering procedure based on hierarchical methods. Associations between cluster membership and socio-economic variables were investigated using multivariable multinomial logistic regression.

**Results:**

A total of 1391 participants were included in analyses and four clusters were identified: (1) “Moderate to high PA, low soft drinkers, low alcohol consumers low SB, non-smokers” (n = 543; 39.0%), (2) “High SB and low fruits and vegetables consumers” (n = 376; 26.8%), (3) “Smokers and alcohol consumers” (n = 247; 17.7%) and (4) “High fruits and vegetable consumers, soft drinkers and low PA” (n = 229; 16.5%). Compared to the healthiest cluster (cluster 1), adolescents from cluster 2&3 (unhealthy clusters) were more likely boys, older, attending vocational high school and not living with their two parents. Adolescents from cluster 4 were more socially advantaged (according to the WHO family affluence scale) than those of cluster 1.

**Conclusions:**

Risk behaviour patterns in adolescents were shown to be clustered in both healthier and less healthy ways, with a complex interplay with socio-economic factors. Adapted to cluster Public health interventions may bring some benefits.

